# The systematic review and meta-analysis of X-ray detective rate of Kashin-Beck disease from 1992 to 2016

**DOI:** 10.1186/s12891-019-2461-z

**Published:** 2019-02-14

**Authors:** Xi Wang, Yujie Ning, Amin Liu, Xin Qi, Meidan Liu, Pan Zhang, Xiong Guo

**Affiliations:** 1 School of Public Health, Xi’an Jiaotong University Health Science Center, Key Laboratory of Trace Elements and Endemic Diseases, National Health and Family Planning Commission, No.76 Yanta West Road, 710061 Xi’an, People’s Republic of China; 2 Xi’an Jiaotong University Global Health Institutes, No.76 Yanta West Road, Xi’an, 710061 People’s Republic of China

**Keywords:** Kashin-Beck disease, Meta-analysis, X-ray detective rate, Correlation analysis

## Abstract

**Background:**

Kashin-Beck disease (KBD) is a serious human endemic chronic osteochondral disease. However, quantitative syntheses of X-ray detective rate studies for KBD are rare. We performed an initial systematic review and meta-analysis to assess the X-ray detective rate of KBD in China.

**Methods:**

For this systematic review and meta-analysis, we searched five databases (PubMed, Web of Science, Chinese National Knowledge Infrastructure (CNKI), WanFang Data and the China Science and Technology Journal Database (VIP))using a comprehensive search strategy to identify studies of KBD X-ray detective rate in China that were published from database inception to January 13, 2018. The X-ray detective rate of KBD was determined via an analysis of published studies using a random effect meta-analysis with the proportions approach. Subgroup analysis and meta-regression were used to explore heterogeneity, and study quality was assessed using the risk of bias tool.

**Results:**

A total of 53 studies involving 14,039 samples with X-ray detective rate in 163,340 observations in total were included in this meta-analysis. These studies were geographically diverse (3 endemic areas). The pooled overall X-ray detective rate for KBD was 11% (95%CI,8–15%;Z = 13.14; *p* < 0.001). The pooled X-ray detective rate estimates were 11% (95%CI, 6–17%; Z = 7.06; *p* < 0.001) for northeast endemic areas, 13% (95%CI, 7–20%; Z = 7.45; *p* < 0.001) for northwest endemic areas, and 8% (95%CI, 5–12%; Z = 7.90; *p* < 0.001) for southwest endemic areas. There was a significant relationship between the survey year and the X-ray detective rate of KBD.

**Conclusions:**

Our systematic review found that the summary estimate of the X-ray detective rate of KBD was 11% and, that KBD X-ray positive rate ranged from 8.00 to 15.00% depending on the study. Further research is required to identify effective strategies for preventing and treating KBD.

**Electronic supplementary material:**

The online version of this article (10.1186/s12891-019-2461-z) contains supplementary material, which is available to authorized users.

## Background

Kashin-Beck disease (KBD) is a serious, endemic chronic osteochondral disease in humans that is distributed from northeast to southwest China, affecting 378 counties in 13 provinces [1]. According to reports from the National Health Commission of China in 2017 [2], the prevalence of KBD in the 13 provinces of China are 1.04% in Hebei, 0.27% in Shanxi, 3.90% in Inner Mongolia, 0.08% in Liaoning, 0.55% in Jilin, 0.49% in Heilongjiang, 0.21% in Shandong, 1.78% in Henan, 5.54% in Sichuan, 2.00% in Tibet, 2.25% in Shaanxi, 1.85% in Gansu and 5.12% in Qinghai Province. KBD is also a major public health problem that results in serious health consequences for patients, including symmetrical enlargement of the phalanges, brachydactyly, joint deformity, and even dwarfism [3], which leads most patients with KBD to partially or completely lose their working capacity and self-care abilities. This outcome not only seriously impacts patient quality of life but also increases the medical burden on society [4].

The diagnosis and degrees of KBD is a crucial first step in the public health approach to confirm this type of osteochondrosis in China. X-ray image, considered as an important diagnostic criterion for KBD, could reflect the damage of articular cartilage before any clinical manifestations. For example, X-ray image can detect the lesions at epiphysis and metaphysis. However, the lack of consensus in diagnosing KBD has resulted in wide variations in reported X-ray detective rate. For example, according to reports of a national survey of KBD prevalence in 2005 [5], National KBD surveillance group found that the X-ray detective rate of KBD was less than 3.00% in the east part of endemic region and was more than 10% in the west part of endemic region, some endemic region in west were 25%. In another analysis of national surveillance on KBD condition from 2000 to 2007, Liu et al. showed that the average X-ray detective rate of KBD in west endemic region has decreased from 21.75% in 2000 to 7.30% in 2007 [6]. Therefore, a number of controversies remain related to estimating the X-ray detective rate of KBD.

To data, few studies have synthesized the results of KBD X-ray detective rate studies. Given the large number of X-ray detective rate studies that have been published in recent decades and the absence of nationwide quantitative estimates of the X-ray detective rate of KBD, it is an opportune time to perform a full systematic review and quantitative analysis of KBD X-ray detective rate.

Here, we report the results of such a study in China to emphasize the need for more accurate nationwide and regional estimates of KBD X-ray detective rate. To analyze the wide variations in X-ray detective rate estimates, we investigated the influence of studies’ demographic and methodological characteristics.

## Methods

### Search strategy and selection criteria

A comprehensive four steps search strategy was used to identify the relevant studies in this systematic review and meta-analysis. The study followed the Preferred Reporting Items for Systematic reviews and Meta-analysis (PRISMA) guidelines [7]. We searched the following academic databases from January 1st 1990 up to January 13th 2018: PubMed, Web of Science, Chinese National Knowledge Infrastructure (CNKI) (articles in Chinese), WanFang Data (articles in Chinese) and the China Science and Technology Journal Database (VIP) (articles in Chinese). We used the following combinations of keywords as the search terms: “ X-ray detective rate ” or “ X-ray ” or “ detective rate ” or “detection rate” and “Kashin-Beck disease” or “KBD” or “endemic osteochondrosis.” The language was limited to English or Chinese. We also reviewed the references cited in the studies and review articles to avoid missing studies. All publications were screened independently based on titles and abstracts, followed by retrieval; the full text publications were screened by two reviewers using the eligibility criteria described below. We searched the scientific literature systematically for observational cross-sectional studies that reported the X-ray detective rate of KBD. We excluded studies that were reviews, conference abstracts, or used qualitative methods only.

### Data extraction and quality assessment

Data were extracted by three reviewers. The following data were extracted:1) study first author, 2) year and areas of publication, 3) number of KBD patients in the sample, 4) X-ray detective rate of KBD in the sample, and 5) age range of the sample. One reviewer crosschecked for accuracy. After removing duplicate studies, two reviewers screened the titles and abstracts using the eligibility criteria, with independent verification by other two reviewers. For suitable studies, we obtained the full text, and scrutinized the text against eligibility criteria, with independent verification by other two reviewers. The study quality was assessed by two reviewers using the standardized Risk of Bias Tool (Additional file 1), which was designed to assess population-based prevalence studies, as part of the data extraction strategy. To assess the risk of bias, the reviewers rated each of the ten items using a dichotomous ratings system with the following categories: low risk and high risk. An overall score was calculated by adding all the items rated as low risk. Thus, higher scores indicated a lower risk of bias and a stronger method quality.

### Data analysis

A random-effect meta-analysis using the proportions approach [8] was used to quantify the X-ray detective rate of KBD. Traditional meta-analysis approaches face the problem that X-ray detective rate proportions approach the limits of 0% or 100%. Thus, a few revised procedures, such as the recently developed metaprop Stata command [8], have been explored to solve this problem. The command metaprop pools proportions and uses the score statistic and the exact binomial method, with the option to incorporate the Freeman-Tukey double arcsine transformation, to compute 95% confidence intervals. A random-effects model within metaprop was chosen due to the considerable heterogeneity across studies in terms of both the measurement of frailty and the samples studied. In random-effects models, the effect sizes of the observed studies are considered to represent a distribution of possible effects; random-effects meta-analysis incorporates both within-study variance and between-study heterogeneity [9]. Studies reporting area estimates were trisected into three groups according to the measurement type, as follows: “northeast,” “northwest” and “southwest.” The X-ray detective rate of KBD was quantified overall and by measurement type. In addition to weighted estimates, the 95% confidence interval (CI) was reported along with z tests (weighted estimate divided by the standard error of the weighted estimate) and associated *p* values as metrics of precision.

Heterogeneity was assessed by using the chi-square test of the Q statistic, which was quantified by the *I*^2^ values. We also calculated *I*^2^ as a “signal-to-noise” ratio of excess dispersion to total dispersion, with values of 25% (indicating that all of the heterogeneity is low), 50% (indicating that all of the heterogeneity is moderate) and 100% (indicating that all of the heterogeneity is high and requires further examination and explanation) [10]. Publication bias and bias associated with small study effects were assessed visually with funnel plots, Egger’s linear regression and Begg’s rank correlation test [11]. To explain the significant observed heterogeneity, stratified analyses and a random-effects meta-regression were performed. Our main factors of interest were the study design (retrospective vs. prospective), geographic region, mean age, study year and study quality score. A sensitivity analysis was also performed by excluding one study at a time to assess whether one or more studies influenced the overall results. *P* ≤ 0.05 indicated statistical significance.

## Results

### Included studies

**Fig. 1:**
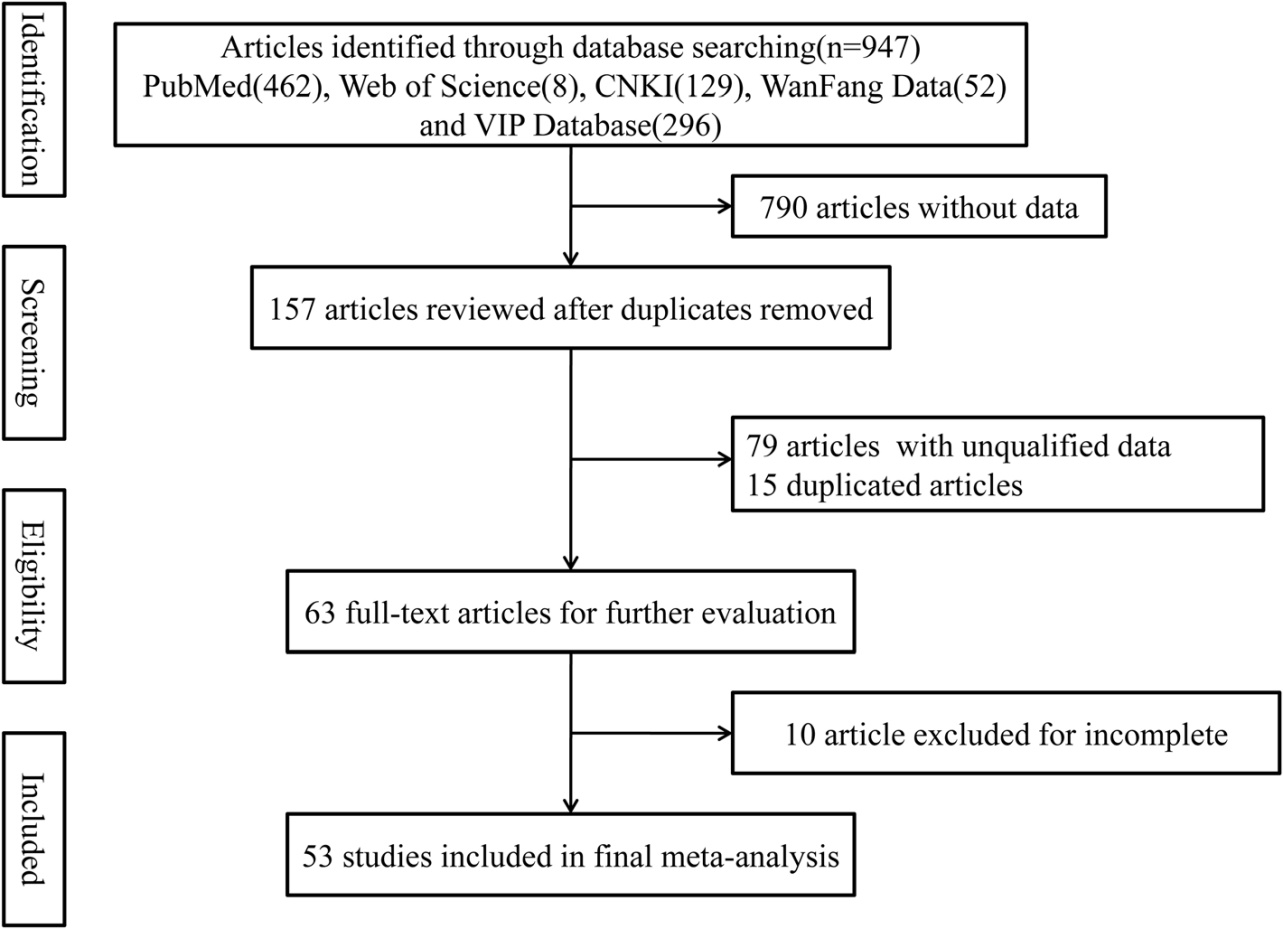
PRISMA flowchart showing the study selection process

The results of study identification, screening, eligibility, and inclusion are shown in the PRISMA flow diagram (Fig. 1). We identified 947 reports, 790 of which were without data, 79 with unqualified data, 10 with incomplete data, and 15 duplicates. Finally, 53 published studies, involving 14,039 samples with X-ray detective rate in 163,340 observations in total, were considered to be eligible and included in the meta-analysis [12–62] (Table 1). The 53 studies selected for the meta-analysis were geographically diverse and included three parts of the KBD endemic area, with 15 studies from the northeast endemic area, 28 studies from the northwest endemic area and 10 studies from the southwest endemic area.

**Table 1:** Selected characteristics of the studies included in this systematic review and meta-analysis

Study	Year	Areas	Case(n)	Total(n)	X-ray detective rate (%)[95%CI]	Age (year)	Study	QS	Comprehensive Preventive Measures
Zhang et al.	2005	Heilongjiang (NE)	270	1699	15.89(14.15,17.63)	7–12	Rep	10	1.Changing grain; 2. Supplemental Se
Su et al.	2012	Jilin (NE)	8	742	1.08 (0.34,1.82)	7–12	Rep	10	1.Changing grain; 2.water improvement
Tao et al.	2012	Inner Mongolia (NE)	54	929	5.81 (4.31,7.32)	7–12	Rep	9	
Xun et al.	2009	Inner Mongolia (NE)	397	2281	17.40 (15.85,18.96)	7–12	Rep	10	1. Changing grain; 2. Changing dietary patterns; 3.Improved economic conditions; 4. water improvement
Xun et al.	2007	Inner Mongolia (NE)	67	871	7.69 (5.92,9.46)	7–12	Rep	10	1. Changing grain;
Yuan et al.	1998	Inner Mongolia (NE)	297	863	34.41 (31.24,37.58)	7–13	Rep	9	
Yuan et al.	1998	Inner Mongolia (NE)	386	1693	22.80 (20.8,24.8)	7–13	Rep	9	1. Changing grain; 2. Supplemental Se
Liu et al.	2001	Inner Mongolia (NE)	172	2498	6.89 (5.89,7.88)	7–13	Pro	10	1. Changing grain
Zhang et al.	1995	Inner Mongolia (NE)	308	687	44.83 (41.11,48.55)	7–12	Rep	10	1. Changing grain
Zhou et al.	2013	Beijing (NE)	25	2055	1.22 (0.74,1.69)	7–12	Rep	10	1. Changing grain; 2. Changing dietary patterns; 3. Improved economic conditions; 4. Collective relocation
Wang et al.	2011	Beijing (NE)	576	5935	9.70 (8.95,10.46)		Rep	8	1. Changing dietary patterns; 2.Improved economic conditions; 3. Collective relocation
Chen et al.	2011	Shandong (NE)	41	3091	1.33 (0.92,1.73)	7–12	Rep	10	1. Water improvement
Yu et al.	2011	Henan (NE)	17	1928	0.88 (0.46,1.30)	7–12	Rep	10	1. Supplemental Se; 2. Changing grain
Yu et al.	2001	Henan (NE)	200	567	35.27 (31.34,39.21)	3–7	Pro	8	1. Supplemental Se
Yang et al.	1994	Henan (NE)	4	155	2.58 (0.08, 5.08)	7–12	Rep	8	1. Supplemental Se
Wang et al.	2011	Shaanxi (NW)	49	259	18.92 (14.15,23.69)	7–16	Rep	9	1. Changing dietary patterns; 2. Supplemental Se; 3. Improved economic conditions
He et al.	2010	Shaanxi (NW)	32	355	9.01 (6.03,12.00)	6–13	Rep	9	1. Changing dietary patterns; 2. Water improvement; 3. Improved economic conditions
Xie et al.	2010	Shaanxi (NW)	1	505	0.20(−0.19,0.59)	7–12	Pro	10	1. Changing grain; 2. Water improvement; 3. Collective relocation
Xie et al.	2008	Shaanxi (NW)	2	610	0.33(−0.13, 0.78)	7–13	Pro	10	1. Changing dietary patterns; 2. Supplemental Se; 3. Water improvement; 4. Improved economic conditions
Lv et al.	2009	Shaanxi (NW)	37	8747	0.42 (0.29, 0.56)	7–13	Rep	10	1. Changing dietary patterns; 2. Supplemental Se; 3. Water improvement; 4. Improved economic conditions; 5. Returning farmland to forest
Xu et al.	2004	Shaanxi (NW)	69	3059	2.26 (1.73, 2.78)	7–12	Rep	10	1. Supplemental Se; 2. Water improvement; 3. Collective relocation
Cao et al.	2004	Shaanxi (NW)	151	944	16.00 (13.66, 18.33)	7–12	Rep	10	1. Supplemental Se
Yang et al.	2005	Shaanxi (NW)	4	110	3.64 (0.14, 7.13)	7–12	Rep	9	1. Changing grain
Wang et al.	2001	Shaanxi (NW)	60	524	11.45 (8.72, 14.18)	7–12	Rep	9	1. Supplemental Se
Chen et al.	2004	Shaanxi (NW)	26	840	3.09 (1.92, 4.27)	7–12	Rep	10	1. Changing grain; 2. Supplemental Se; 3.Water improvement
Sun et al.	1992	Shaanxi (NW)	299	970	30.82 (27.92, 33.73)	3–13	Rep	9	1. Supplemental Se
Xu et al.	2009	Shaanxi (NW)	487	2844	17.12 (15.74, 18.51)	7–12	Rep	10	1. Supplemental Se
Xie et al.	2011	Shaanxi (NW)	38	2248	1.69 (1.16, 2.22)	7–12	Pro	10	1. Supplemental Se
Lv et al.	2002	Shaanxi (NW)	441	3324	13.27 (12.11, 14.42)	6–13	Rep	10	1. Supplemental Se; 2.Water improvement
Zhang et al.	2002	Shaanxi (NW)	500	2107	23.73 (21.91, 25.55)	7–12	Rep	10	1. Supplemental Se
Yi et al.	2016	Shaanxi (NW)	6	1744	0.34 (0.07, 0.62)	7–12	Rep	9	1. Changing grain; 2. Water improvement
Cui et al.	2013	Gansu (NW)	83	2254	3.68 (2.90, 4.46)	7–12	Rep	10	1. Supplemental Se
Ge et al.	2008	Gansu (NW)	345	4367	7.90 (7.10, 8.70)	7–12	Rep	10	1. Supplemental Se
Liu et al.	2007	Gansu (NW)	56	567	9.88 (7.42, 12.33)	7–12	Rep	10	1. Supplemental Se; 2. Changing grain
Zhang et al.	2014	Gansu (NW)	31	1019	3.04 (1.99, 4.10)	7–12	Rep	10	1. Supplemental Se
Bai et al.	2002	Gansu (NW)	484	802	60.22 (56.83, 63.61)	3–13	Pro	10	1. Supplemental Se
Luo et al.	2012	Gansu (NW)	21	610	3.44 (2.00, 4.89)	7–14	Rep	10	1. Supplemental Se
Li et al.	2004	Qinghai (NW)	383	1194	32.08 (29.43, 34.72)	7–12	Rep	10	1. Supplemental Se; 2. Water improvement
Ding et al.	2001	Qinghai (NW)	566	1290	43.88 (41.68, 46.58)	7–12	Rep	9	
Ding et al.	2003	Qinghai (NW)	291	1446	20.12 (18.06, 22.19)	7–12	Rep	10	1. Water improvement; 2. Supplemental Se; 3. Changing dietary patterns
Li et al.	2006	Qinghai (NW)	2228	5122	43.50 (42.14, 44.86)	7–12	Rep	9	1. Water improvement; 2. Supplemental Se
Bao et al.	1998	Qinghai (NW)	195	436	44.72 (40.06, 49.39)	7–12	Rep	10	
Li et al.	2002	Qinghai (NW)	168	407	41.28 (36.49, 46.06)	7–12	Rep	10	
Zhao et al.	2016	Tibet (SW)	1388	43,034	3.23 (3.06, 3.39)	7–12	Rep	9	1. Supplemental Se; 2. Changing grain; 3. Collective relocation; 4. Improved economic conditions
Zhao et al.	2011	Tibet (SW)	15	431	3.48 (1.75, 5.21)	7–12	Pro	10	
Gong et al.	2004	Tibet (SW)	73	611	11.95 (9.38, 14.52)	4–12	Pro	10	1. Supplemental Se
A et al.	2015	Sichuan (SW)	182	14,189	1.28 (1.10, 1.47)	7–12	Rep	10	1. Supplemental Se
Wang et al.	2005	Sichuan (SW)	551	3149	17.50 (16.17, 18.82)	7–12	Rep	10	1. Supplemental Se
Zhang et al.	2009	Sichuan (SW)	24	1243	1.93 (1.17, 2.70)	7–12	Rep	10	1. Supplemental Se; 2. Changing dietary patterns
Li et al.	2003	Sichuan (SW)	314	1600	19.63 (17.68, 21.57)	7–12	Rep	10	1. Supplemental Se; 2. Changing dietary patterns
Deng et al.	2008	Sichuan (SW)	675	8689	7.77 (7.21, 8.33)	7–13	Rep	10	1. Improved economic conditions; 2. Changing dietary patterns
Hunag et al.	2010	Sichuan (SW)	373	13,472	2.77 (2.49, 3.05)	7–12	Rep	10	1. Supplemental Se; 2. Collective relocation; 3. Changing grain
Deng et al.	1999	Sichuan (SW)	569	2224	25.58 (23.77, 27.40)	7–12	Rep	10	1. Improved economic conditions

QS: quality score; Rep: retrospective cohort study; Pro: prospective cohort study. NE: northeast; NW: northwest; SW: southwest

### Meta-analysis

**Fig. 2:**
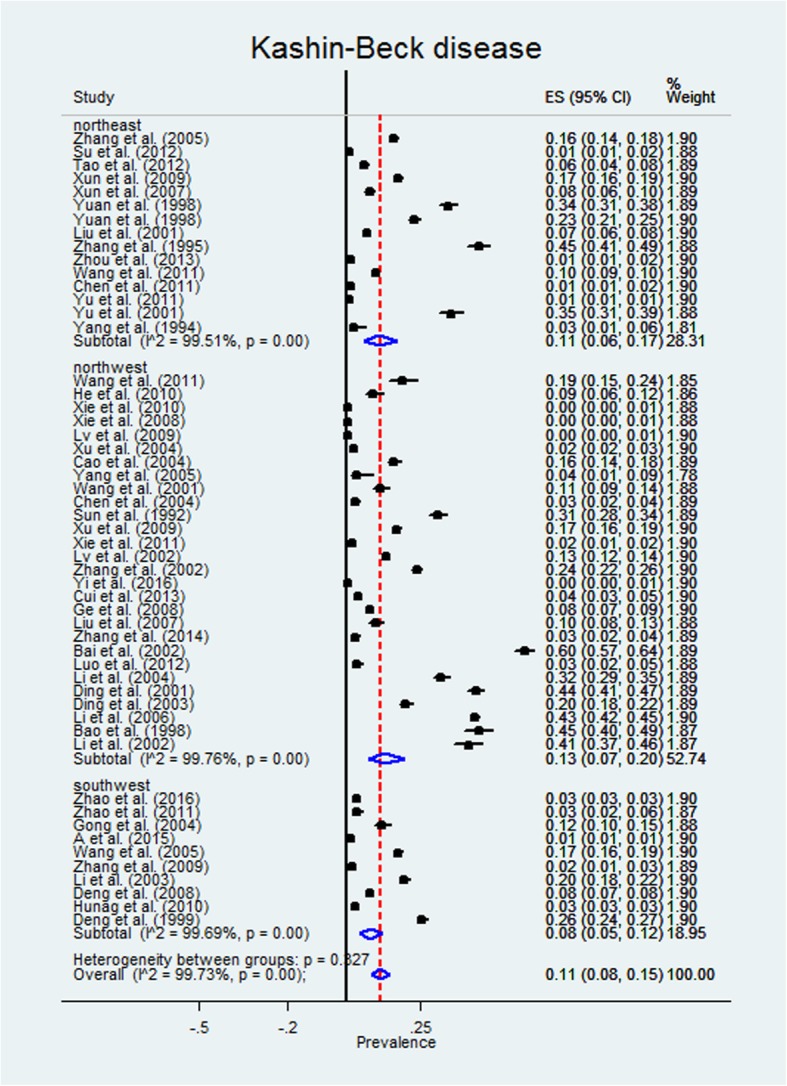
Forest plot of pooled estimated X-ray detective rate of KBD with 95% CI

**Fig. 3:**
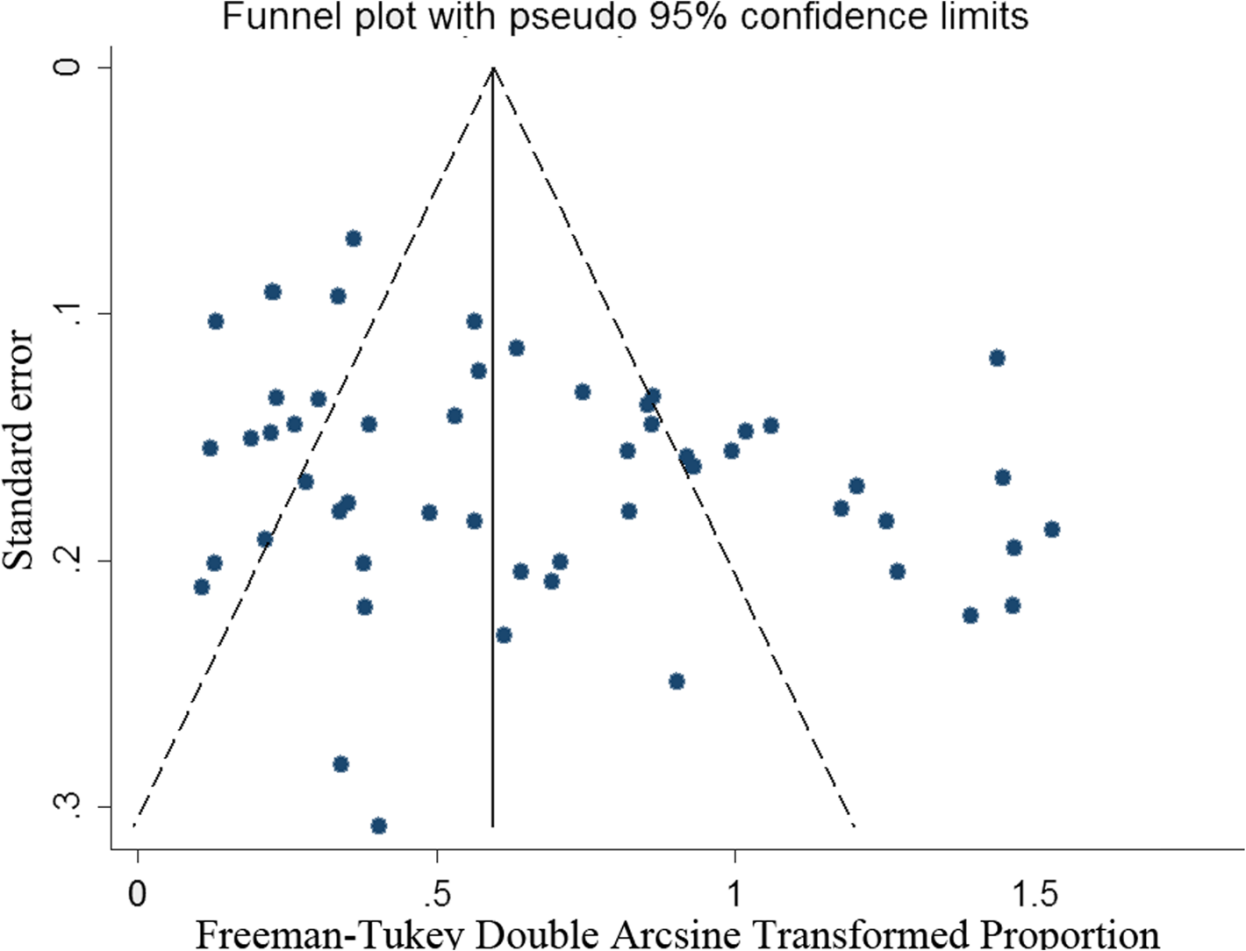
Funnel plot of the 35 studies included in the meta-analysis

The overall estimated X-ray detective rate of KBD was 11% (95%CI,8–15%;Z = 13.14; *p* < 0.001). Heterogeneity statistics (Q = 19,151.88; *I*^2^ = 99.73%) indicated that there was significant and substantive heterogeneity in the X-ray detective rate of KBD across studies (Fig. 2). To confirm the stability and liability of the meta-analysis, a sensitivity analysis was performed by recalculating the pooled KBD X-ray detective rate when any single study was deleted. The results show that the pooled X-ray detective rate (14%; 95%CI, 11–18%) did not change significantly. A visual inspection of the funnel plot revealed slight asymmetry (Fig. 3), but both Begg’s test (Z = − 1.14, *P* = 0.259) and Egger’s test (t = 0.32, *P* = 0.747) showed no potential risk of publication bias.

### Subgroup results

To provide a range of KBD X-ray detective rate estimates in the KBD endemic areas, estimates were stratified by the northeast, northwest, and southwest endemic areas. The estimated X-ray detective rate of KBD in the northeast was 11% (95%CI, 6–17%; Z = 7.06; *p* < 0.001). The estimated X-ray detective rate of KBD in the northwest was 13% (95%CI, 7–20%; Z = 7.45; p < 0.001). The estimated X-ray detective rate of KBD in the southwest was 8% (95%CI, 5–12%; Z = 7.90; p < 0.001) (Fig. 2).

### Meta-regression

**Fig. 4:**
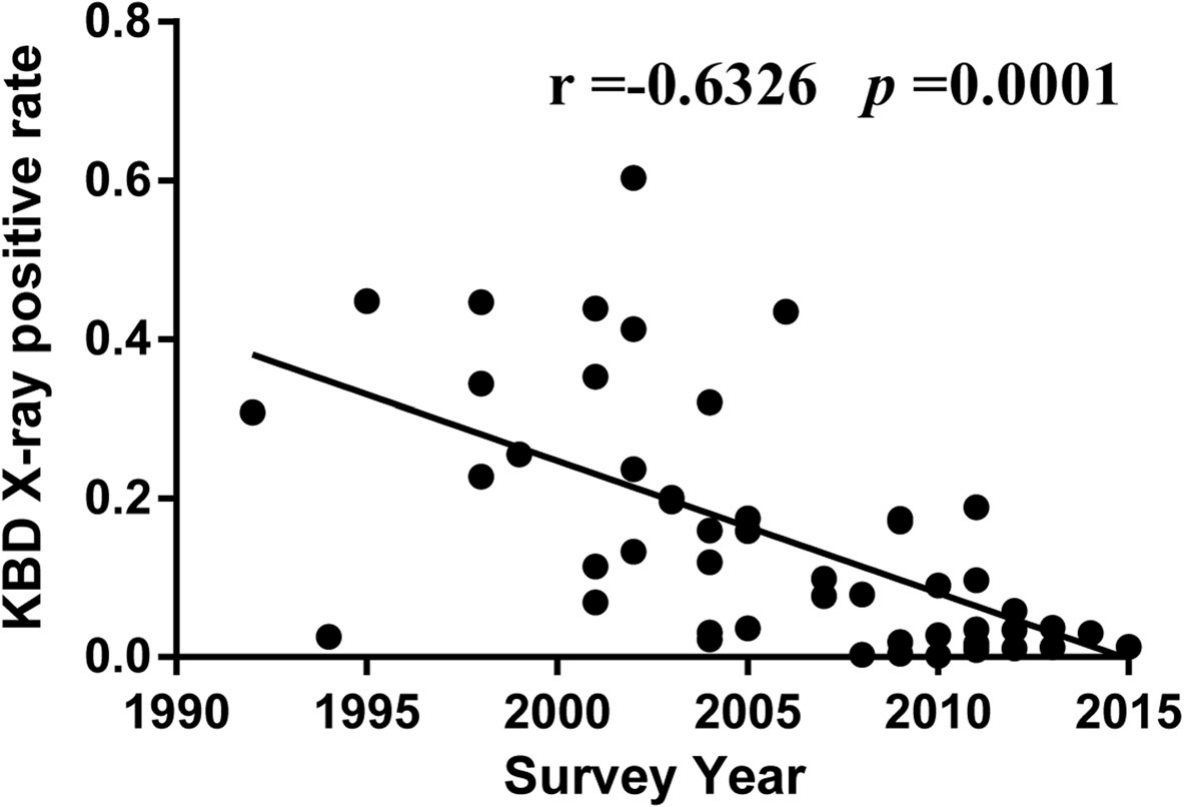
The correlation between KBD X-ray detective rate and percentage of survey year

Meta-regression was performed to explore potential sources of heterogeneity. Survey year, age, study quality score and KBD endemic areas were tested as potential sources of heterogeneity. Only survey year (t = − 5.82; *P* = 0.000) was significantly associated with the detected heterogeneity (Table 2). We therefore further tested the correlation between KBD X-ray detective rate and potential sources of heterogeneity. There was a negative correlation between KBD X-ray detective rate and survey year(r = − 0.6326, *P* = 0.0001) (Fig. 4 and Additional file 2: Table S1).

**Table 2:** Results of meta-regression for the prevalence of Kashin-Beck disease. QS: quality score; EA: endemic area

Covariate	coefficient	95%CI	t-value	*P*-value
Survey year	−0.0164	−0.02203, −0.10937	−5.82	0.000
age	−0.0315	−0.07480, 0.01173	−1.43	0.153
QS	−0.0091	−0.06712, 0.04890	−0.31	0.758
EA	0.0064	−0.04004, 0.05286	0.27	0.787

## Discussion

Despite substantial heterogeneity among the studies included in our study, we generated a precise estimate of the X-ray detective rate of KBD based on data from 53 published articles that involved 14,039 samples with X-ray detective rate in 163,340 observations in total in China via a systematic review and meta-analysis of studies published from 1992 to 2016.

In the report of a national survey of Kashin-Beck disease prevalence in 2005 [5], the X-ray detective rates of 11 spots were not more than 3% in the east part of endemic areas. The X-ray detective rates more than 10% in 5 of the 10 spots in the western parts, with the highest one being 25%(Qinghai Province). The X-ray detective rates were 0.96 and 5.04% in Sichuan, 5.56 and 6.02% in Shaanxi, 10.53% and 0 in Inner Mongolia, 14.71 and 16.98% in Gansu, 12.10 and 25% in Qinghai. The average detective rate was 5.21% in all spots. In our meta-analysis, the average detective rate was 11%, and the X-ray detective rate of KBD in the northeast was 11, 13% in the northwest and 8% in the southwest by subgroup analysis. In our meta-analysis, the X-ray detective rates data were selected from the studies published from 1992 to 2016, which were more comprehensive and persuasive than the survey in one year. But the trend of the two results above is consistent, the highest X-ray detective rate was northwest.

Moreover, the X-ray detective rate of KBD may be reflective of other mechanisms that are beneficial for preventing KBD. Although the average the X-ray detective rates of KBD was 5.21% in the report of a national survey of Kashin-Beck disease prevalence in 2005, the X-ray detective rates of KBD in some endemic areas in the northwest remained as high as 25%, and the high X-ray detective rates of KBD demonstrated that this disorder remains an important problem for some underdeveloped endemic areas, In addition, KBD is more common in some endemic areas than previously thought, which has important implications for researchers focusing on children with KBD.

To our knowledge, this review was the first to perform a systematic review and meta-analysis reporting KBD X-ray detective rates estimates in China. The aim of this study was to explore the overall X-ray detective rate of KBD in China from 1992 to 2016.

In this study, differences among X-ray detective rates estimates in different KBD endemic areas in China may be caused by intertwined factors, such as demographic and socioeconomic characteristics, life styles, and health and nutrition status. Interestingly, there was a negative correlation between KBD 1992 to 2016 and survey year, indicating that the mean X-ray detective rate decreased progressively. The comprehensive preventive measures such as improving drinking water and grain, relocating residents in endemic areas and improving balanced nutrition in KBD endemic areas may be the main reasons for the decreased X-ray detective rates of KBD, specifically the comprehensive preventive measures used in most of endemic areas. In Yi’s study, local residents ate grain purchased outside of the endemic areas instead of grain grown in endemic areas, and the condition of the drinking water was also improved [41]. In another study, the comprehensive preventive measures against KBD included supplemental Se, water supply improvements and altered dietary patterns [50]. For decades, it has been recommended that patients with KBD and residents living in endemic areas should prevent and cure KBD with supplemental Se, improved drinking water conditions, and altered the grains and improving dietary patterns. The measures above did decrease the risk of KBD in endemic areas. Recently, researchers have been developing and improving new diagnostic techniques for KBD and exploring the etiology and pathogenesis of KBD, which are the key to controlling and even eliminating KBD.

This systematic review and meta-analysis included 53 published articles that involved 14,039 samples with X-ray detective rate in 163,340 observations in total in China, and there is no potential risk of publication bias. Meta-regression analyses were conducted to find potential sources of heterogeneity. In addition, a sensitivity analysis was performed to confirm the robustness of our results. Nevertheless, several limitations should be acknowledged when interpreting the findings of our study. First, because KBD occurs primarily in China, the 53 articles included in this meta-analysis were all from Asia, which could lead to a bias in statistical analyses and the estimation of X-ray detective rate on a global scale due to the variability of the sample size and the unbalanced distribution of the studies. Second, most of the studies (45/53) included in this study were retrospective observational studies, which are considered to provide moderate evidence. Thus, the conclusion drawn in this analysis is restricted by this study type. Finally, heterogeneity was observed in the study, which was not surprising as heterogeneity often exists in such meta-analyses of overall X-ray detective rate. Although subgroup and meta-regression analyses suggested that geographic region and survey year could explain a portion of the observed heterogeneity, the remaining heterogeneity among the studies was not explained by the examined variables.

## Conclusions

In conclusion, our meta-analysis found that the estimated the overall X-ray detective rate of KBD was 11% and ranged from 8 to 15% depending on the study. Further research is required to identify effective strategies for preventing and treating KBD.

## Additional files

Additional file 1: Risk of Bias Tool: criteria for assessment of quality. (DOCX 13 kb)

Additional file 2: **Table S1.** The correlation between the mean prevalence and potential sources. QS: quality score. (DOCX 14 kb)

## Abbreviations

CI: Confidence Interval
CNKI: Chinese National Knowledge Infrastructure
KBD: Kashin-Beck Disease
PRISMA: Systematic reviews and Meta-analysis
