# Supplementary material for: The systematic review and meta-analysis of X-ray detective rate of Kashin-Beck disease from 1992 to 2016
Source: BMC Musculoskelet Disord. 2019 Feb 14;20:78. doi: 10.1186/s12891-019-2461-z (PMC6376664; doi:10.1186/s12891-019-2461-z)
Supplement: Supplementary file 1 — Risk of Bias Tool: criteria for assessment of quality. (DOCX 13 kb) [file 12891_2019_2461_MOESM1_ESM.docx]

Risk of Bias Tool: criteria for assessment of quality

**External validity (maximum score=4)**

1 Was the study’s target population a close representation of the national population (subnational or city) in relation to relevant variables such as age, sex, occupation?

(Yes: low risk=1 point; no: high risk=0 points)

2 Was the sampling frame a true or close representation of the target population?

(Yes: low risk=1 point; no: high risk=0 points)

3 Was some form of random selection used to select the sample, or was a census undertaken? (Yes: low risk=1 point; no: high risk=0 points)

4 Was the likelihood of non-response bias minimal?

(Yes: low risk=1 point; no: high risk=0 points)

**Internal validity (maximum score=6)**

1 Were data collected directly from the subjects (as opposed to a proxy)?

(Yes: low risk=1 point; no: high risk=0 points)

2 Was an acceptable case definition used in the study?

(Yes: low risk=1 point; no: high risk=0 points)

3 Was the study instrument that measured the parameter of interest shown to have reliability and validity (if necessary)?

(Yes: low risk=1 point; no: high risk=0 points)

4 Was the same mode of data collection used for all subjects?

(Yes: low risk=1 point; no: high risk=0 points)

5 Was the length of the shortest prevalence period for the parameter of interest appropriate?

(Yes: low risk=1 point; no: high risk=0 points)

6 Were the numerator(s) and denominator(s) for the parameter of interest appropriate? (Yes: low risk=1 point; no: high risk=0 points)
