# Supplementary material for: The systematic review and meta-analysis of X-ray detective rate of Kashin-Beck disease from 1992 to 2016
Source: BMC Musculoskelet Disord. 2019 Feb 14;20:78. doi: 10.1186/s12891-019-2461-z (PMC6376664; doi:10.1186/s12891-019-2461-z)
Supplement: Supplementary file 2 — Table S1. The correlation between the mean prevalence and potential sources. QS: quality score. (DOCX 14 kb) [file 12891_2019_2461_MOESM2_ESM.docx]

| Covariate | Survey year | QS |
| --- | --- | --- |
| r | -0.6326 | -0.301 |
| *P* | 0.0001 | 0.1273 |

**Table S1**. The correlation between the X-ray detective rate and potential sources. QS: quality score.
